# Incidence and risk factors of gonococcal urethritis reinfection among Thai male patients in a multicenter, retrospective cohort study

**DOI:** 10.1038/s41598-021-02398-6

**Published:** 2021-11-26

**Authors:** Monai Meesaeng, Boonsub Sakboonyarat, Supitchaya Thaiwat

**Affiliations:** 1 Department of Medicine, Phramongkutklao Hospital, Bangkok, 10400 Thailand; 2 Department of Military and Community Medicine, Phramongkutklao College of Medicine, Bangkok, 10400 Thailand; 3 Division of Dermatology, Department of Medicine, Phramongkutklao Hospital, Bangkok, 10400 Thailand

**Keywords:** Epidemiology, Medical research, Risk factors, Infectious diseases, Urogenital diseases, Public health

## Abstract

Gonococcal urethritis (GU) is the second most common sexually transmitted infection (STI). Epidemiologic studies of the situation of GU reinfection and its related risk factors among patients with a history of GU in Thailand remain somewhat limited. A hospital-based retrospective cohort study was conducted between January 1, 2010 and December 31, 2020 to determine the incidence and risk factors of GU reinfection among male patients visiting in Royal Thai Army (RTA) Hospitals. A total of 2,465 male patients presenting a history of GU was included in this study. In all, 147 (6.0%; 95% CI 5.1–6.9) male patients presented GU reinfection, representing an incidence rate of 1.3 (95% CI 1.1–1.5) per 100 person-years. The independent risk factors for GU reinfection were age < 30 years (AHR 1.7; 95% CI 1.0–2.8), number of sexual partners equal to 2 (AHR 3.4; 95% CI 1.0–11.2), $\ge$ 3 (AHR 5.6; 95% CI 2.7–11.6), and participants residing in the north (AHR 4.1; 95% CI 2.3–7.5) and northeast regions (AHR 2.1; 95% CI 1.1–3.9). Incidence of GU reinfection among male patients visiting RTA Hospitals was significantly high among younger aged patients, especially in the north and northeast regions. Multiple sex partners played a major role in GU reinfection. Effective STI prevention programs should be provided to alleviate reinfection and its complications.

## Introduction

Gonococcal urethritis (GU) is the second most common sexually transmitted infection (STI) and has been increasing both among males and females, affecting public health and economics worldwide^1^. Gonorrhea is caused by *Neisseria gonorrhoeae* infection; a columnar-type epithelial infection at the genital organs among both males and females^2,3^. When the disease is untreated, the infection would invade adjacent organs causing prostatitis, or posterior urethritis leading to infertility^2,4,5^.

Globally in 2018, the World Health Organization reported that the estimated cases of GU involved 87 million patients and about 27 million were in the Asia Pacific^6^. In Thailand, the data for the population aged 15–24 years demonstrated that *Neisseria gonorrhoeae* infection increased from 38.7 per 100,000 population in 2014 to 68.1 in 2017^7^. A related population-based study in the US reported that the cumulative incidence of GU reinfection was 5%^8^; additionally, a wide range of incidence rates was found for reinfection from 2.3 to 4.3 per 100 person years^9,10^. Sexual behaviors including having multiple sexual partners remain risk factors for GU infection^11,12^. Moreover, a related study reported that GU reinfection was more common in young male populations^8^. Additionally, GU infection also related to other STIs including non-gonococcal infection and HIV^13,14^.

The related evidence demonstrated that the prevalence of gonococcal transmission in Thailand has been rising continuously. However, the essential information of GU reinfection and its related risk factors among patients with a history of GU in Thailand remain somewhat limited. Furthermore, a recent study reported that a history of gonococcal infection was an independent risk for antimicrobial-resistant *Neisseria gonorrhoeae*^15^*.* Essential information is required to focus disease prevention and control strategies. Attenuating the risk for gonococcal infection will help to reduce the transmission and to alleviate its complications. Therefore*,* the investigators aimed to determine the incidence and risk factors for GU reinfection among men visiting military hospitals in all regions of Thailand.

## Methods

### Study designs and subjects

A hospital-based retrospective cohort study was conducted between January 1, 2010 and December 31, 2020 to determine the incidence and risk factors of gonococcal urethritis reinfection among male patients visiting in the Royal Thai Army (RTA) Hospitals. In Thailand, 37 RTA Hospitals are located nationwide in five regions, i.e., central, north, northeast, south and Bangkok. Each RTA Hospital provides medical care for both military personnel and civilians. As a tertiary (standard/advanced) hospital, the RTA Hospitals, selected to be the study sites, were located in five provinces of five regions including Phramongkutklao Hospital in Bangkok, Anandamahidol Hospital in Lop Buri (central), Fort Somdej Phra Naresuan Hospital in Phitsanulok (north), Fort Suranaree Hospital in Nakhon Ratchasima (northeast), and Fort Wachirawut Hospital in Nakhon Si Thammarat (south). The eligible criteria of participants comprised male patients aged at least 20 years presenting a history of GU. GU was determined according to the International Classification of Diseases, Tenth Revision codes (ICD-10) in A540, A541, A548 or A549, presented in medical records^16^. At each RTA Hospital, the internist would be available at the STI clinic to provide care. Typically, male patients with suspected GU infection would access care at STI clinics. History taking and physical examination would be performed by the internist. Patients presenting gram-negative intracellular diplococci on microscopic examination of a smear of mucopurulent urethral exudate received a diagnosis of GU.

### Data collection

Patient information was retrieved from the database of STI clinics. In all, 2,465 male patients in five hospitals were included in this study. A standardized case report form (CRF) was used to collect data of patients from medical records, including demographic characteristics, age, residential area, occupation, marital status, number of lifetime sexual partners, history of GU diagnosis, history of other STIs and sexual behaviors. The data in the medical records of patients were retrieved and filled in the CRF by trained registered nurses in five hospitals and sent to the data management unit at Phramongkutklao College of Medicine in Bangkok.

### Ethics consideration

The study was reviewed and approved by the Institutional Review Board, Royal Thai Army Medical Department in compliance with international guidelines such as the Declaration of Helsinki, the Belmont Report, CIOMS Guidelines and the International Conference on Harmonization of Technical Requirements for Registration of Pharmaceuticals for Human Use—Good Clinical Practice (ICH-GCP) (Approval number R126h/62). Due to the retrospective cohort study design, a waiver of documentation of consent was used; the waiver for consent was granted by the Institutional Review Board, Royal Thai Army Medical Department.

### Statistical analysis

All analyses were conducted using StataCorp, 2021, *Stata Statistical Software: Release 17*, College Station, TX, USA: StataCorp LLC. Demographic characteristics were analyzed using descriptive statistics. Continuous data were presented as mean and standard deviation while percentage was used for categorical data. The incidence rates per 100 person-years of observation for GU reinfection was presented. Reinfection was defined as the first episode of GU diagnosed at least 14 days after a previously recorded infection for patients during the study period^10^. The person-times of observation of the participants were calculated as the duration between a previously treated GU and the next reinfection at the end of the study period, whichever occurred first.

Patients having no record of reinfection were assumed to be free of subsequent infection throughout the follow-up period. Participants’ follow-up was right censored December 31, 2020. Multivariate cox regression analysis was performed to obtain the adjusted hazard ratio (AHR) and 95% confidence interval (95% CI) of the risk factors related to the first episode of GU reinfection during the study period. A *p*-value less than 0.05 was considered statistically significant.

## Results

### Characteristics of study participants

A total of 2,465 male patients, presenting a history of GU from five regions of Thailand, was included in this study. All the subjects were males aged 27.2 ± 7.9 years. The majority of patients were from a hospital in Bangkok (37.9%). In terms of occupation, 42.0% of participants comprised government officers. The median number of lifetime sexual partners was 1.0. Participants with a history of consistent condom use among participants was 6.2%. A history of other STIs and sexual behaviors is presented in Table 1.

**Table 1:** Baseline characteristics of participants (N = 2465).

Demographic data	n (%)
**Age (years)**	27.2 ± 7.9
< 20	59 (2.4)
20–29	1893 (76.8)
30–39	360 (14.6)
40–49	68 (2.8)
≥ 50	85 (3.4)
**Occupation**	
Government officers	1035 (42.0)
Conscripts	236 (9.6)
Employee	195 (7.9)
Students	94 (3.8)
No^a^	42 (1.7)
Undetermined	863 (35.0)
**Marital status**	
Single	2257 (91.6)
Married	208 (8.4)
**Hospitals**	
Bangkok	934 (37.9)
North	580 (23.5)
Northeast	526 (21.3)
Central	306 (12.4)
South	119 (4.8)
**Scheme**	
Universal Coverage	1573 (63.8)
Government Officer	518 (21.0)
Social Security	86 (3.5)
Others	288 (11.7)
**Number of lifetime sexual partners**	
Median (min–max)	1.0 (1.0–10.0)
**History of HIV testing**	
No	2089 (84.7)
Yes	376 (15.3)
**HIV status**	
Positive	4 (0.01)
Negative	372 (99.9)
**History of HbsAg testing**	
No	2264 (91.8)
Yes	201 (8.2)
**HbsAg status**	
Positive	6 (3.0)
Negative	195 (97.0)
**History of anti-HCV testing**	
No	2307 (93.6)
Yes	158 (6.4)
**Anti-HCV status**	
Positive	0 (0.0)
Negative	158 (100.0)
**History of VDRL testing**	
No	1878 (76.2)
Yes	587 (23.8)
**VDRL status**	
Non-Reactive	580 (97.6)
Reactive	7 (2.4)
**History of having sex with another man**	
No	186 (97.3)
Yes	5 (2.7)
**History of consistent condom use**	
No	437 (93.8)
Yes	29 (6.2)

^a^No occupation was defined by the medical record reported that the participants have no occupation.

### Incidence and risk factors of GU reinfection among male patients visiting RTA hospitals

A total of 147 (6.0%; 95% CI 5.1–6.9) male patients presented GU reinfection, representing an incidence rate of 1.3 (95% CI 1.1–1.5) per 100 person-years. The univariate and multivariate cox regression analysis identifying risk factors for GU reinfection are shown in Table 2. The independent risk factors for GU reinfection were age < 30 years (AHR 1.7; 95%CI 1.0–2.8), number of lifetime sexual partners equal to 2 (AHR 3.4; 95%CI 1.0–11.2), ≥ 3 (AHR 5.6; 95%CI 2.7–11.6), and participants residing in the north (AHR 4.1; 95%CI 2.3–7.5) and northeast regions (AHR 2.1; 95%CI 1.1–3.9), adjusting for age, occupations, regions and number of lifetime sexual partner.

**Table 2:** Risk factors for gonococcal urethritis reinfection among male patients visiting in the RTA hospitals.

Risk factors	No. of incidents	Person-year of observation	Incidence rate per 100 person-years	Crude hazard ratio (95% CI)	*p*-value	Adjusted hazard ratio (95% CI)	*p*-value
**Total**	**147**	**11,440.1**	**1.28 (1.09–1.51)**				
**Age (years)**							
≥ 30	20	3187.50	0.63	1		1	
< 30	127	8252.59	1.54	2.28 (1.40–3.69)	< 0.001	1.67 (1.01–2.79)	0.049
**Occupation**							
Government officers	53	5107.90	1.04	1		1	
No	2	198.19	1.01	1.14 (0.28–4.70)	0.854	1.33 (0.27–6.49)	0.725
Students	3	474.66	0.63	0.76 (0.23–2.46)	0.652	0.38 (0.11–1.32)	0.127
Employee	12	1031.16	1.16	1.31 (0.70–2.45)	0.400	1.19 (0.62–2.30)	0.603
Conscripts	10	1071.19	0.93	0.99 (0.50–1.95)	0.975	0.84 (0.30–2.33)	0.732
Others	67	3556.99	1.88	1.80 (1.25–2.59)	0.002	0.76 (0.44–1.31)	0.323
**Marital status**							
Single	137	10,321.73	1.33	1			
Married	10	1118.37	0.90	0.71 (0.37–1.35)	0.293		
**Regions**							
Bangkok	42	4825.74	0.88	1		1	
North	59	2254.82	2.62	2.78 (1.86–4.14)	< 0.001	4.12 (2.28–7.45)	< 0.001
Northeast	29	2361.70	1.23	1.36 (0.85–2.18)	0.204	2.11 (1.13–3.94)	0.019
Central	14	1385.57	1.02	1.10 (0.60–2.02)	0.748	1.94 (0.78–4.82)	0.152
South	3	612.30	0.49	0.56 (0.17–1.79)	0.326	0.77 (0.19–3.13)	0.718
**Number of lifetime sex partners**							
1	135	11,256.50	1.20	1		1	
2	3	42.47	7.07	6.35 (2.02–19.93)	0.002	3.37 (1.01–11.23)	0.048
≥ 3	9	138.59	6.50	4.73 (2.41–9.30)	< 0.001	5.6 (2.70–11.60)	< 0.001
**History of consistent condom use**							
No	39	1836.99	2.12	1			
Yes	2	122.94	1.63	0.84 (0.20–3.50)	0.812		

Multivariate cox regression analysis (adjusted for age, occupation, regions, number of lifetime sex partners.

## Discussion

Our data demonstrated that the incidence rate of GU reinfection among male patients visiting RTA Hospitals between 2010 and 2020 was 1.3 per 100 person-years. GU reinfection was significantly related to personal characteristics and a history of multiple sexual partners. Estimates of the incidence of GU remain limited in Southeast Asia, including Thailand. To our knowledge, this constitutes the first report on the incidence of GU reinfection among Thai men visiting hospitals located in all regions.

The cumulative incidence of GU reinfection among men in this study accounting for 6.0% and may be comparable to the cumulative incidence of those among men in a population-based study in California, USA. (5%)^8^. However, longitudinal studies conducted in public STI clinics in Baltimore, USA, and in Alberta, Canada indicated incidence was 4.3 and 2.3 reinfections per 100 person years, respectively, indicating the incidence rate of reinfection in the present study was relatively low^9^. The reported GU reinfection in the present study was based on medical records in hospitals; thus, only male patients with GU visiting the hospitals would be recorded while others visiting STI clinics were excluded from this study. According to this reason, the incidence of GU reinfection in the present study may have been underestimated.

We found that younger age male patients (< 30 years old) were highly related to GU reinfection. Similarly, related reports conducted in the USA and Canada showed that patients aged less than 25 years were independently at risk of increased reinfection^9,10^. Furthermore, one related study in the New Zealand health care setting demonstrated that patients aged less than 20 years were more likely to have a potential risk for *Neisseria gonorrhoeae* reinfection^17^. This finding may be explained by younger sexually active males using condoms inconsistently^18^, leading to STIs including GU, nonGU and HIV^12,19,20^. Regarding one related report among young Thai men from 2008 to 2009^12^, male patients having multiple sexual partners indicated a risk behavior for STIs and tended to be a risk for GU reinfection. The finding was consistent with related studies of Rangsin et al. revealing young males having multiple sexual partners ≥ 4 were more likely to have STIs^12,13^. In terms of occupation, our finding reported no significant evidence of any association between occupation and GU reinfection. However, a related study in Thailand demonstrated that unemployed young men were associated with STIs such as *Chlamydia trachomatis* infection^12^.

In terms of geographic region of the participants, the results of the study participants may not represent the incidence rate of GU reinfection among all male patients in these regions, especially in Bangkok where several clinics and hospitals are available. Male patients with GU may not receive care at the same hospital. Therefore, the results reflected the situation of those among male patients vising RTA Hospitals located in each region of Thailand. The incidence rate of GU reinfection among participants was significantly high in the north (2.6 per 100 person-years) and northeast (1.2 per 100 person-years). These regions also experienced the highest epidemic of HIV/AIDS and other STIs such as *Chlamydia trachomatis* and Hepatitis C^7,20^. Likewise, related studies in Thailand demonstrated a high prevalence of STIs among young Thai men residing in north^12^ and northeast regions^20^. One of the explanations for this finding is that GU reinfection may be a proxy indicator for other STIs in these regions. Furthermore, the knowledge of STIs such as gonococcal infection may be limited; some people did not know the mode of transmission or prevention for gonococcal infection^21,22^.

We found that male patients vising RTA Hospitals reporting a history of consistent condom use was 6.3%. Our data suggested that effective interventions such as 100% condom use should be implemented among young Thai men, especially in the north and the northeast, to alleviate the transmission of *Neisseria gonorrhoeae, Chlamydia trachomatis* and HIV*,* and its complications including periurethral abscesses, postinflammatory urethral strictures and pelvic inflammatory diseases^23,24^. In addition, modifiable risk behaviors such as having multiple sexual partners should be diminished. Furthermore, the system for active STIs case detection, STIs education, motivation to seek diagnosis and treatment in youth and general populations should be established^7^.

Limitations involving these data must be considered. First, this constituted a hospital-based retrospective cohort study; thus, we were unable to obtain confirmation by cultures of the reported *Neisseria gonorrhoeae* infection. However, we attempted to define GU according to the ICD-10 appearing in medical records. Although this was a hospital-based study, the results of the present study corresponded to those of the report by the Department of Disease Control, Ministry of Public Health demonstrating a continuous increase in GC infection cases in the north and northeast regions^7^. Second, we intended to determine the situation of GU reinfection; unfortunately, the data of the other sites of gonococcal infection, including rectum and throat, were not collected in this study. Therefore, the rates for gonococcal reinfection in these other sites were unreported. Third, male patients presenting a history of GU may not revisit the same hospital; they may visit STI clinics located at the provincial public health office. Hence, the incidence of GU reinfection may have been underestimated. This issue may be associated with the loss to follow-up and might have introduced selection bias. However, in Thailand, the universal health coverage scheme was implemented since 2002^25^; therefore, the Thai population could access care at registered hospitals. RTA Hospitals are under the government administration and provide care for military personnel and civilians residing in the area. Because of their health scheme attached to the specific hospital, most patients would receive care at the same RTA Hospital. Since this comprised a retrospective cohort study, we lacked the opportunity to explore risk behaviors including history of sex in exchange for gifts/money and history of sex with a female sex worker among these subjects; only data available in medical records were analyzed. However, the case report form was completed by well-trained registered nurses using a standard protocol. Finally, a small number in some variable categories was included; thus, the association between well-known risk factors such as history of other STIs, history of consistent condom use, sexual behaviors and outcome could not be presented.

## Conclusion

In conclusion, the incidence of GU reinfection among male patients visiting RTA Hospitals was significantly high among younger aged patients vising the five hospitals, especially in the north and northeast. Multiple sex partners played a major role in GU reinfection in this population. Effective STI prevention programs should be implemented to attenuate reinfection and its complications.

## Data Availability

The datasets generated during and/or analyzed during the current study are not publicly available because the data set contains sensitive identifying information; thus, ethical restrictions are required concerning the data set. However, the data set are available from the corresponding author on reasonable request.

## Abbreviations

GU: Gonococcal urethritis
STI: Sexually transmitted infection
HIV: Human immunodeficiency virus
ICD-10: The International Classification of Diseases, tenth revision codes
HR: Hazard ratio
AHR: Adjusted hazard ratio
95%CI: 95% Confidence interval
